# The role of genome-wide DNA methylation and polymorphisms in periodontitis etiology: A narrative review

**DOI:** 10.17305/bb.2025.12646

**Published:** 2025-07-03

**Authors:** Elena Jovanova, Angela Angjelova, Alessandro Polizzi, Gaetano Isola

**Affiliations:** 1 University Dental Clinical Center St. Pantelejmon, Skopje, Faculty of Dentistry, Ss. Cyril and Methodius University in Skopje, Skopje, North Macedonia; 2 Department of General Surgery and Surgical-Medical Specialties, School of Dentistry, University of Catania, Catania, Italy; 3 International Research Center on Periodontal and Systemic Health; “PerioHealth”, University of Catania, Catania, Italy

**Keywords:** DNA methylation, periodontitis, genome-wide association studies, GWAS, genetic polymorphisms, epigenetic mechanisms

## Abstract

Periodontitis is a multifactorial inflammatory disease influenced by genetic, epigenetic, and environmental factors. Recent advancements in genomic and epigenomic research have highlighted the role of genetic polymorphisms and genome-wide DNA methylation in its pathogenesis. DNA methylation regulates gene expression, affecting immune responses and inflammatory pathways, while genetic polymorphisms may predispose individuals to altered host-microbial interactions and increased susceptibility to periodontal destruction. Recent studies have identified promising periodontal biomarkers, including specific genetic and epigenetic markers, that may aid in early diagnosis, risk assessment, and monitoring of disease progression. This narrative review synthesizes current evidence on the genetic and epigenetic mechanisms involved in the etiology of periodontitis, with a focus on genome-wide DNA methylation and genetic polymorphisms. It also explores their potential implications for disease pathogenesis, diagnostics, and therapeutic strategies. Future research directions include integrative multi-omics approaches to better understand the complex interplay between genetic, epigenetic, and environmental factors. Such efforts aim to support the development of personalized therapeutic strategies. Overall, this review underscores the critical role of genetic and epigenetic mechanisms in the pathogenesis of periodontitis and emphasizes the need to translate these findings into clinical practice through molecular diagnostics and personalized treatment approaches.

## Introduction

Periodontitis is a chronic, multifactorial inflammatory condition caused by a dysbiotic biofilm, which, if not appropriately and proactively treated, can lead to a progressive disruption of supporting periodontal tissues and ultimately result in tooth loss [1]. Pathogenic bacteria present in the plaque biofilm continuously attack the host, activating an immune response that may gradually cause tissue damage due to inflammation. However, the presence of pathogenic subgingival bacteria does not typically lead to periodontal destruction in most cases. Although bacteria are crucial in the onset of periodontitis, the quantity of plaque and specific bacterial species do not necessarily correlate with the disease’s severity [2].

Individuals may exhibit a dose-dependent response to bacterial exposure, influencing their susceptibility to periodontitis. Most individuals are resistant to the disease and do not develop periodontitis. The pathophysiology of periodontitis, like that of other complex diseases, is driven by multiple biological pathways that ultimately result in similar clinical manifestations [3]. Complex diseases are typically polygenic, involving variations in multiple genes, each contributing a small effect and relative risk to the disease process. These genes are considered disease-modifying, especially when periodontitis coexists with systemic diseases [4]. Several aspects of the inflammatory and immune response implicated in the development of periodontitis have a well-defined genetic basis. Genetic predisposition is believed to influence the progression of periodontitis, particularly in its aggressive and rapidly advancing forms [5]. A significant increase in scientific publications has suggested associations between genetic polymorphisms and various medical conditions, particularly chronic immune and inflammatory disorders. This growing body of knowledge indicates that most diseases, including periodontitis, have a genetic foundation [6]. The disease often exhibits a bidirectional relationship with genetic factors, alongside environmental and behavioral influences, which play a role in disease initiation in susceptible individuals and the rate of its progression [7]. Given the known impact of these factors and systemic health on the epigenome, as well as the established role of external exposures in shaping disease predisposition, both epigenetic factors and genetic polymorphisms are significant in the pathobiology of periodontitis [8, 9].

In contrast to the genome, which remains consistent across all cells and throughout an individual’s life, the epigenome is dynamic and varies among different cells and tissues. Epigenetics is an emerging field of science that focuses on changes in gene expression occurring without modifications to the underlying DNA sequence, responding to alterations in the cellular microenvironment and external influences associated with inflammation and disease risk. These changes can lead to the silencing or overexpression of specific genes, producing different molecular outcomes [9–11]. The primary mechanisms of epigenetic regulation include DNA methylation, small non-coding RNAs (ncRNAs), and histone modification [12, 13]. Research on gene methylation in the pathology of periodontitis may provide valuable insights for a more comprehensive understanding of the disease, potentially aiding in the development of effective therapies [14]. Several studies have investigated global DNA methylation patterns in periodontitis, suggesting that various methylation changes play a crucial role in the disease’s pathogenesis [15].

A substantial body of literature has examined the impact of gene variants, including polymorphisms, on host immune responses and the pathogenesis of periodontitis [16]. Specific alterations in the genetic code can lead to changes in the function or secretion of encoded proteins, potentially contributing to increased disease severity or greater susceptibility to the disease [17].

This review aims to provide an overview of current research on the role of genome-wide DNA methylation and genetic polymorphisms in the pathogenesis of periodontitis, emphasizing their significance in disease mechanisms, advanced precision diagnostics, the development of targeted therapies, and ultimately, the improvement of long-term outcomes in periodontal care.

To synthesize this information comprehensively and focus on relevant findings, we conducted a targeted literature search in PubMed and Scopus using keywords such as “periodontitis,” “DNA methylation,” “genetic polymorphisms,” “GWAS,” “EWAS,” and “epigenetics.” Studies were selected based on methodological rigor, sample size, relevance to human periodontitis, and their contributions to elucidating genetic and epigenetic mechanisms. Preference was given to peer-reviewed research that provides clinically or biologically meaningful insights into the etiology, pathogenesis, and potential diagnostic or therapeutic applications related to periodontal disease.

## Genetic polymorphisms in periodontitis

### Key susceptibility genes and polymorphisms associated with periodontitis

Genetic polymorphisms are variations at specific loci within the genome that affect more than 1% of the population. These polymorphisms can alter the structure or expression of encoded proteins, resulting in modifications to both innate and adaptive immune responses [18]. Additionally, certain gene polymorphisms may confer protection against specific diseases, including periodontitis, by influencing the immune system’s response to pathogens [19]. Genetic variations that modulate the efficacy of cellular and humoral immune responses impact an individual’s risk of developing periodontitis [17]. The immune system is widely recognized as playing a significant role in the pathogenesis of periodontitis. Numerous genes are believed to contribute to the development of periodontitis in periodontal tissues, including those that regulate the expression of interleukin (IL)-1, IL-6, tumor necrosis factor (TNF)-α and its receptors, IL-10, selenoprotein S, Fc-γ receptor, CD14 molecule, toll-like receptors (TLRs), caspase recruitment domain 15, and the vitamin D receptor [20].

### Genome-wide association studies (GWAS) and their contributions

GWAS have proven to be a powerful tool for uncovering genetic variants associated with complex human diseases and traits. Numerous GWAS have been conducted to elucidate the genetic underpinnings of periodontitis [21]. Recently, GWAS has emerged as the primary method for analyzing genetic polymorphisms, with many studies focusing on periodontal disease. In an early investigation, Schaefer et al. [22] identified a significant association between aggressive periodontitis (AP) and the single nucleotide polymorphism (SNP) rs1537415 in the glycosyltransferase gene *GLT6D1*. More recent studies have reported associations between periodontal disease and polymorphisms in the *SIGLEC5* gene, which encodes sialic acid-binding immunoglobulin-like lectin 5 [23, 24]. The integration of bioinformatics and GWAS has facilitated the extensive identification of SNPs associated with periodontitis across diverse population groups. One study found that SNPs in the *NPY*, *IL37*, and *NCR2* genes were associated with increased susceptibility to moderate or severe periodontitis, while a variant in *TLR9* was linked to a reduced risk of developing severe disease. These SNPs exhibited sex- and smoking-dependent effects on periodontitis, further supporting the role of genetic predisposition in the disease’s pathogenesis [25].

**Figure 1.**
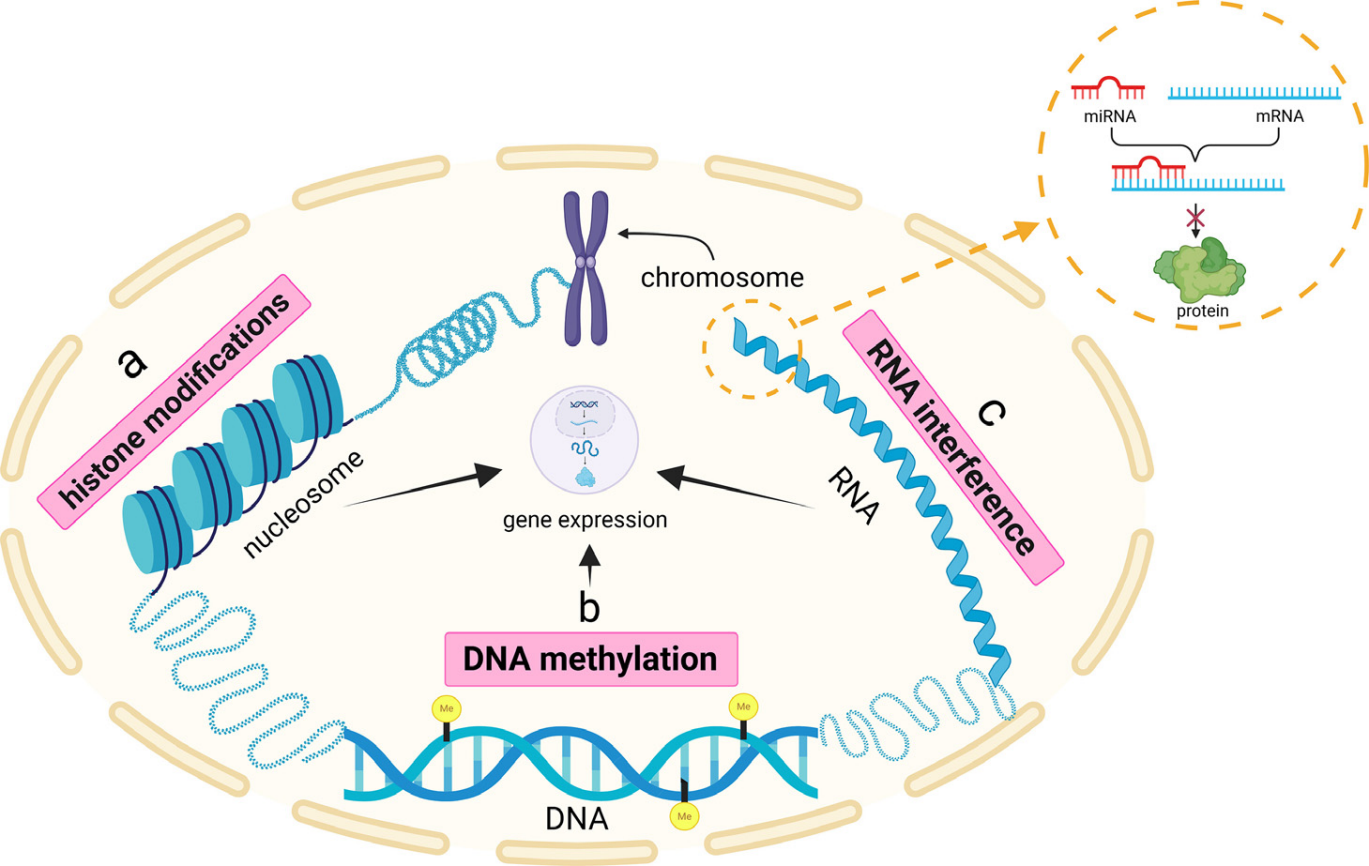
**Schematic representation of the epigenetic mechanisms.** Epigenetic modifications driven by chemical changes to DNA and chromatin remodeling via DNA methylation, histone modifications and by of small ncRNAs in response to dynamic environmental conditions. (A) Histone modications; (B) DNA methylation; (C) Non-coding RNAs. Adapted from [121], with permission under the terms of the Creative Commons Attribution (CCBY) license^©^.

### Functional implications of genetic polymorphisms in periodontal disease pathogenesis

The primary antibody subclass produced in response to infection of the periodontium by pathogenic microorganisms is IgG2. Elevated levels of these antibodies have been detected in the serum of patients with localized AP compared to those with generalized periodontitis, suggesting that higher antibody levels may play a role in mitigating disease severity [26, 27]. Significant differences in IgG2 antibody production have been observed across racial and individual groups, with enhanced production linked to the presence of the n+ allele in the gene encoding the antibody heavy chain (γ2 locus), commonly referred to as the Gm allele [27, 28]. Genetic differences in the production of IL-1 family cytokines may account for the varying severity levels observed in periodontal disease. Some identified polymorphisms are thought to contribute to individual variations in cytokine production that correlate with disease extent [29]. Furthermore, cytokines activate multiple signaling pathways that regulate osteoclastic activity and alveolar bone resorption. Prior research has indicated that polymorphisms in cytokine genes may be associated not only with susceptibility to periodontal disease but also with its progression, intensity, and clinical outcomes [30]. Studies suggest that TNF-α plays a critical role in the pathogenesis and progression of periodontal disease, potentially contributing to its development [31, 32]. Dysregulated cytokine gene expression may perpetuate the persistent cycles of tissue inflammation observed in these disorders [32]. Kornman et al. [33] were among the first to identify genetic markers associated with periodontal disease. They investigated several polymorphisms in the *IL-1* gene and found a correlation between *IL-1* gene variants and the severity of periodontal disease in non-smokers, differentiating between mild and severe cases. These findings established a foundation for subsequent genetic studies on periodontal disease, employing various methodological approaches. Identifying genetic risk factors associated with periodontal disease is crucial for developing effective prevention and treatment strategies [30].

## Epigenetic mechanisms in periodontits

Epigenetic factors are inheritable alterations to the genome that can influence gene expression, potentially contributing to disease development. These modifications are particularly relevant in chronic inflammatory conditions, such as periodontal diseases, where they facilitate microbial persistence or enable microbial damage, thus supporting the ‘hit-and-run’ infectious mechanism that results in prolonged pathogen disruption of the host genome [34]. Epigenetic modifications are driven by mechanisms such as chemical changes to DNA and chromatin remodeling (e.g., via DNA methylation and histone modifications), as well as the actions of small ncRNAs [35]. These changes enable the genome to adjust its transcriptional activity in response to dynamic environmental conditions [36] (Figure 1).

**Table 1:** DNA methylation changes and their implications in periodontitis

**Study**	**Mechanism**	**Finding**
Barros et al., 2014 [46]	DNA methylation	Methylation alterations in chemokine and cytokine genes in epithelial cells from periodontitis sites
Amormino et al., 2013 [44]	DNA methylation	*TLR2* promoter hypermethylation associated with increased periodontal pocket depth
Yin et al., 2011 [48]	DNA methylation	Gingival epithelial cells showed hypermethylation of *IL12A, TLR2, GATA3*, and hypomethylation of *ZNF287* and *STAT5A*
Almiñana-Pastor et al., 2019 [10] Stefani et al., 2013 [8] Schulz et al., 2016 [47]	DNA methylation	*IL-6*, *IL-8*, *IL-10*, *INF-γ*, and *IL-17* potentially linked to host immune responses to periodontal pathogens

### Overview of epigenetic regulation

#### DNA methylation

Within the cell nucleus, DNA is organized into a chromatin structure, tightly wrapped around histone proteins. These histones can undergo post-translational modifications such as acetylation and methylation. The specific patterns of these modifications influence chromatin accessibility and modulate transcription factor binding, thereby regulating gene expression initiation and contributing to distinct cellular responses [37, 38]. The most prominent examples of epigenetic modification are DNA methylation, which generally represses gene expression and involves the addition of a methyl group to cytosine residues followed by guanine or adenine in the DNA sequence [34]. This extensively studied epigenetic process is recognized for its contribution to disease development, including cancer, and its essential role in maintaining normal cellular functions [12]. DNA methylation represents an epigenetic mechanism that is both heritable and potentially reversible, subject to modulation by external environmental agents, such as tobacco use, as well as internal factors like infection and inflammation. These influences can induce dynamic changes in epigenetic regulation throughout an individual’s lifespan, enabling phenotypic plasticity in response to varying stimuli. This adaptability underscores the potential for implementing precision medicine strategies that incorporate both genomic and epigenomic profiles [34]. Additionally, these changes may serve as potential biomarkers for the early stages of cellular transformation [39]. DNA can be chemically altered through the addition of methyl groups to cytosine bases within CpG sites—regions where cytosine and guanine are linked by a phosphate group, commonly referred to as CpG islands [40]. This methylation process is mediated by a group of DNA methyltransferases (DNMTs), which transfer a methyl group from S-adenosylmethionine (SAM) to the fifth carbon of a cytosine base, resulting in the formation of 5-methylcytosine (5mC) [41]. Hypomethylation of CpG sites within gene promoters is generally associated with enhanced transcriptional activity, whereas hypermethylation in the same regions is commonly correlated with gene silencing and reduced transcriptional output [42]. *In vitro* evidence indicates that distinct epithelial cell types, such as oral keratinocytes, immortalized human keratinocytes (HaCaT), and gingival epithelial cells, experience significant downregulation of *DNMT1* expression when exposed to *Fusobacterium nucleatum*, *Porphyromonas gingivalis*, or isolated lipopolysaccharide (LPS) [34].

Previous studies investigating DNA methylation at pre-selected gene loci have shown that methylation levels in inflammatory gene promoters, such as interferon-gamma (IFN-γ), *TLR2*, and TNFα, are altered in periodontitis tissues, often correlating inversely with gene expression [43–45]. Barros and Offenbacher [46], applying laser capture microdissection, detected widespread methylation alterations in chemokine and cytokine genes in gingival epithelial cells from periodontitis sites. Hypomethylation of promoter regions in genes encoding pro-inflammatory cytokines—including *IL-6*, *IL-8*, IFN-γ, and *IL-17*—has been observed in human gingival biopsy samples and is potentially linked to host immune responses to periodontal pathogens [8, 10, 47]. Moreover, upon stimulation with the periodontal pathogen *P. gingivalis*, gingival epithelial cells exhibited promoter-specific hypermethylation of *IL12A*, *TLR2*, and *GATA3*, alongside hypomethylation of *ZNF287* and *STAT5A* genes [48, 49]. *TLR2* promoter hypermethylation was found to be associated with increased periodontal pocket depth [44]. Differential CpG methylation of the thioredoxin gene, involved in *IL-1β* driven innate immune responses, has been identified in periodontitis patients [34].

Given the multifactorial etiology of periodontal disease, which is heavily influenced by environmental exposures and modifiable systemic conditions, integrative approaches may enhance the understanding and management of the disease. In this context, the role of DNA methylation in the progression of periodontitis has been examined (Table 1).

#### Histone modifications

Posttranslational histone modifications significantly influence chromatin structure and gene transcription through the addition of methyl groups to lysine or arginine residues on histones H3 and H4, facilitated by histone methyltransferases and demethylases. Lysine modifications on histones are the most prevalent, with histone H3 being the most extensively modified [50, 51]. Lysine methylation occurs in mono-, di-, or trimethylation forms, and the specific methylation state correlates with changes in gene transcription activity. Recent studies have expanded the identification of lysine methylation sites associated with transcriptional activation (e.g., H3K4, H3K36, and H3K79) and repression (e.g., H3K9, H3K27, and H4K20) [52]. Among these, methylation sites H3K4, H3K9, and H3K27 are the most extensively studied and recognized for their impact on dental tissues [53, 54]. Limited research has explored histone modifications in response to bacterial LPSs. Larsson et al. reported that LPS promotes H3 methylation and acetylation of H3 and H4 in B cells, while other studies have demonstrated that histone deacetylase (HDAC) inhibitors, which enhance histone acetylation, protect against LPS-induced bone resorption [55, 56]. Alterations in histone methylation status regulate periodontal gene expression and significantly impact periodontal development, health, and treatment [51].

#### ncRNAs

ncRNAs are RNA molecules that do not encode proteins but regulate gene expression and cell differentiation at genomic and chromosomal levels. They are primarily classified into microRNAs (miRNAs) and long ncRNAs (lncRNAs) based on their length. miRNAs are short, evolutionarily conserved RNA molecules ranging from 17 to 25 nucleotides, derived from longer precursor transcripts, while lncRNAs are linear RNA transcripts exceeding 200 nucleotides. Microarray analysis has revealed differential expression of 159 miRNAs and 8925 lncRNAs, indicating their potential involvement in the pathogenesis and progression of periodontitis [57]. In inflammatory diseases such as periodontitis, lncRNAs are increasingly implicated in modulating immune responses, cytokine expression, and the activity of signaling pathways involved in tissue destruction and repair [58, 59]. These lncRNAs have been shown to regulate various cellular processes in periodontal cells, including osteogenic differentiation, inflammatory responses, cell proliferation, autophagy, and apoptosis [60]. Several differentially expressed miRNAs were identified, most of which are functionally associated with inflammatory responses and cellular homeostasis. Moreover, circulating miRNAs in serum may serve as potential biomarkers for various diseases [61]. Real-time PCR confirmed increased expression levels of miR-664a-3p, miR-501-5p, and miR-21-3p, suggesting their roles as serum-based biomarkers for diagnosing periodontitis [62]. A comprehensive meta-analysis identified miRNA-146a and miRNA-142-3p as significantly associated with periodontitis, indicating their potential role in the disease’s pathogenesis [63]. Notably, miRNA-146a is one of the most extensively studied miRNAs in periodontitis, underscoring its relevance in periodontal inflammation and immune regulation [64]. It has been reported to negatively regulate the innate immune response, with significantly higher levels observed in chronic periodontitis patients. Additionally, miRNA-146a expression is inversely correlated with pro-inflammatory cytokines TNF-α and IL-6 [65] (Table 2).

**Table 2:** Histone methylation and non-coding RNA changes and their implications in periodontitis

**Modification type**	**Gene/Region**	**Modification**	**Associated finding**
Histone methylation	H3 (*H3K4, H3K36 H3K79*)	Mono-, Di-, Trimethylation	Transcriptional activation in periodontal tissues
Histone methylation	H3 (*H3K9, H3K27*), H4 (*H4K20*)	Mono-, Di-, Trimethylation	Transcriptional repression in periodontal tissues
Histone acetylation	H3, H4	Acetylation	LPS-induced acetylation in B cells; HDAC inhibitors protect against bone resorption
ncRNA	miR-664a-3p, miR-501-5p, miR-21-3p	Differential expression	Potential serum biomarkers for periodontitis
ncRNA	miRNA-146a	Increased expression	Regulates immune response in periodontitis; inversely correlated with TNF-α, IL-6
ncRNA	Various lncRNAs	Differential expression	Involved in immune response and tissue repair in periodontitis

Abbreviations: ncRNA: Non-coding RNA; lncRNAs: Long non-coding RNAs; miRNA: MicroRNA; LPS: Lipopolysaccharide; HDAC: Histone deacetylase.

**Table 3:** Genome-wide DNA methylation studies related to periodontitis

**Locus/Gene**	**Type**	**Finding**
MTND1P5	GWAS locus (chr8)	Identified as a novel risk locus for aggressive and chronic periodontitis
SHISA9	GWAS locus (chr16)	Newly linked to periodontitis at genome-wide significance
WHAMM	GWAS-associated gene	SNPs near this gene associated with periodontitis
ZNF804A	GWAS-linked CpG site	Methylation at this site is partially genetically controlled

Abbreviation: GWAS: Genome-wide association studies.

### Genome-wide DNA methylation studies in periodontitis

Given the substantial heterogeneity and limited replication of SNPs identified through GWAS in periodontitis, integrating epigenome-wide DNA methylation analyses may enhance the interpretation of genetic findings by elucidating regulatory mechanisms mediating genetic susceptibility to periodontal disease [66]. A recent meta-analysis of GWAS identified two novel risk loci: one located within the *MTND1P5* pseudogene on chromosome 8 and another near the *SHISA9* gene on chromosome 16. These loci reached genome-wide significance, providing new genetic insights into disease susceptibility [67]. Methylation at several CpG sites is partially under genetic control, consistent with previous GWAS findings, which identified SNPs in or near genes such as *WHAMM*. These results suggest that inherited genetic variants may interact with environmentally responsive epigenetic modifications to influence disease susceptibility [68]. However, replication of these findings across independent cohorts remains limited, reflecting challenges related to sample size heterogeneity, phenotype definitions, and study populations [67]. To advance understanding and clinical utility, future research should emphasize large, multi-ethnic cohorts and integrative multi-omics approaches to validate and expand these genetic and epigenetic associations [69] (Table 3).

### Impact of epigenetic modifications on immune response and inflammation

Epigenetic modifications, including DNA methylation and histone alterations, play a pivotal role in modulating immune responses and inflammatory pathways by regulating gene expression in immune cells [70]. Changes in the epigenetic landscape of innate immune genes can disrupt normal inflammatory responses [46].

LPS, a major component of outer membrane vesicles from Gram-negative bacteria, is recognized as a potent immune activator. Exposure to purified LPS can elicit a robust pro-inflammatory response and, at elevated concentrations, may result in septic shock [71]. Specific periodontal pathogens and their components, such as LPSs, have been implicated in inducing epigenetic alterations within periodontal tissues. *In vitro* studies indicate that oral bacteria induce cell type-specific epigenetic changes, with methylation modifications affecting gene regulation contingent upon the cell’s function and its interaction with particular pathogens.

Elevated levels of DNA methylation in the promoter region of a gene are typically associated with decreased gene expression, whereas promoter hypomethylation is generally linked to increased transcriptional activity [72]. Alterations in DNMT activity have been documented in cells exposed to either whole bacterial lysates or isolated LPSs [34]. Research indicates that *P. gingivalis* LPS induces hypermethylation of the *RUNX* gene in periodontal cells, suggesting that targeting epigenetic modifications may facilitate the treatment of periodontal disease. Runt-related transcription factor 2 (*RUNX2*) is a crucial transcription factor associated with osteoblast differentiation, and the inhibition of osteoblastic differentiation correlates with reduced *RUNX2* expression [73].

A significant suppression of nuclear DNMT-3a (*DNMT3A*) mRNA expression has also been observed in HaCaT cells following stimulation with *Porphyromonas gingivalis* LPS, indicating a similar regulatory response [74]. Kim et al. [75] noted that selective modulation of the immune response alters the activity of DNA (cytosine-5)-methyltransferase 1 and HDACs, both of which are critical regulators of DNA methylation and histone modification processes.

## Interplay between genetic and epigenetic factors

### Gene-environment interactions and their role in periodontal susceptibility

Gene-environment interactions encompass the combined effects of genetic predispositions and environmental exposures on susceptibility to human diseases [76]. Numerous exogenous and endogenous risk factors engage in prolonged interactions with the host organism, often spanning several years, prior to the onset of chronic degenerative diseases. Tobacco use and chronic inflammation, particularly driven by specific bacterial pathogens, are significant contributors to the development of periodontitis. A central pathogenic mechanism involves oxidative damage, supported by the identification of mitochondrial DNA lesions within the gingival tissue of individuals diagnosed with the disease [77]. While microbial and environmental factors are acknowledged as key initiators and modulators of periodontal disease progression, individual susceptibility to these conditions varies markedly and is predominantly determined by the host’s immune response to periodontal pathogens [78]. The biofilms associated with gingivitis and periodontitis consist of localized, complex communities of multiple microbial species that demonstrate strong resistance to both antimicrobial therapies and the host’s immune system. Recent advancements in the epidemiological study of periodontal disease and risk factor analysis have established that systemic risk factors within the host significantly influence individual variability in disease onset, progression rate, and severity [79].

### Influence of lifestyle factors on epigenomic modifications

Fraga et al. [80] suggested that variations in epigenetic modifications observed between monozygotic twins are likely induced by environmental factors such as tobacco exposure and nutritional differences. Seddon et al. [81] proposed that behavioral and nutritional factors, including vitamin D, betaine, and methionine, can induce epigenetic modifications that impact disease progression. Various nutritional elements, including folate, vitamin B12, and vitamin A, have been shown to influence epigenetic changes. Folate, a water-soluble vitamin found in dark green leafy vegetables, strawberries, and asparagus, is extensively studied in relation to cancer due to its role as a methyl group donor in DNA methylation processes. Consequently, reduced folate intake correlates with decreased levels of DNA methylation [82]. The progression of attachment loss may be influenced by epigenetic mechanisms, as evidenced by Ohi et al. [83], who reported elevated methylation levels in the *COL1A1* gene—a key structural protein in the periodontal ligament (PDL)—in older individuals compared to their younger counterparts.

Multiple studies have established a connection between oral microbiota and host genetic factors. Specific polymorphisms in the *IL-6* gene identified in individuals with severe periodontitis have been consistently associated with the presence of *Aggregatibacter actinomycetemcomitans* and *Porphyromonas gingivalis* [84]. These findings suggest that a distinct genotype may be linked to a particular subgingival bacterial profile, influencing the host’s susceptibility to periodontal disease. Furthermore, epigenetic modifications may modulate periodontal pathogens, as altered DNA methylation has been shown to reduce the invasive potential of *Aggregatibacter actinomycetemcomitansin* oral epithelial cells, thereby diminishing its virulence [85].

**Figure 2.**
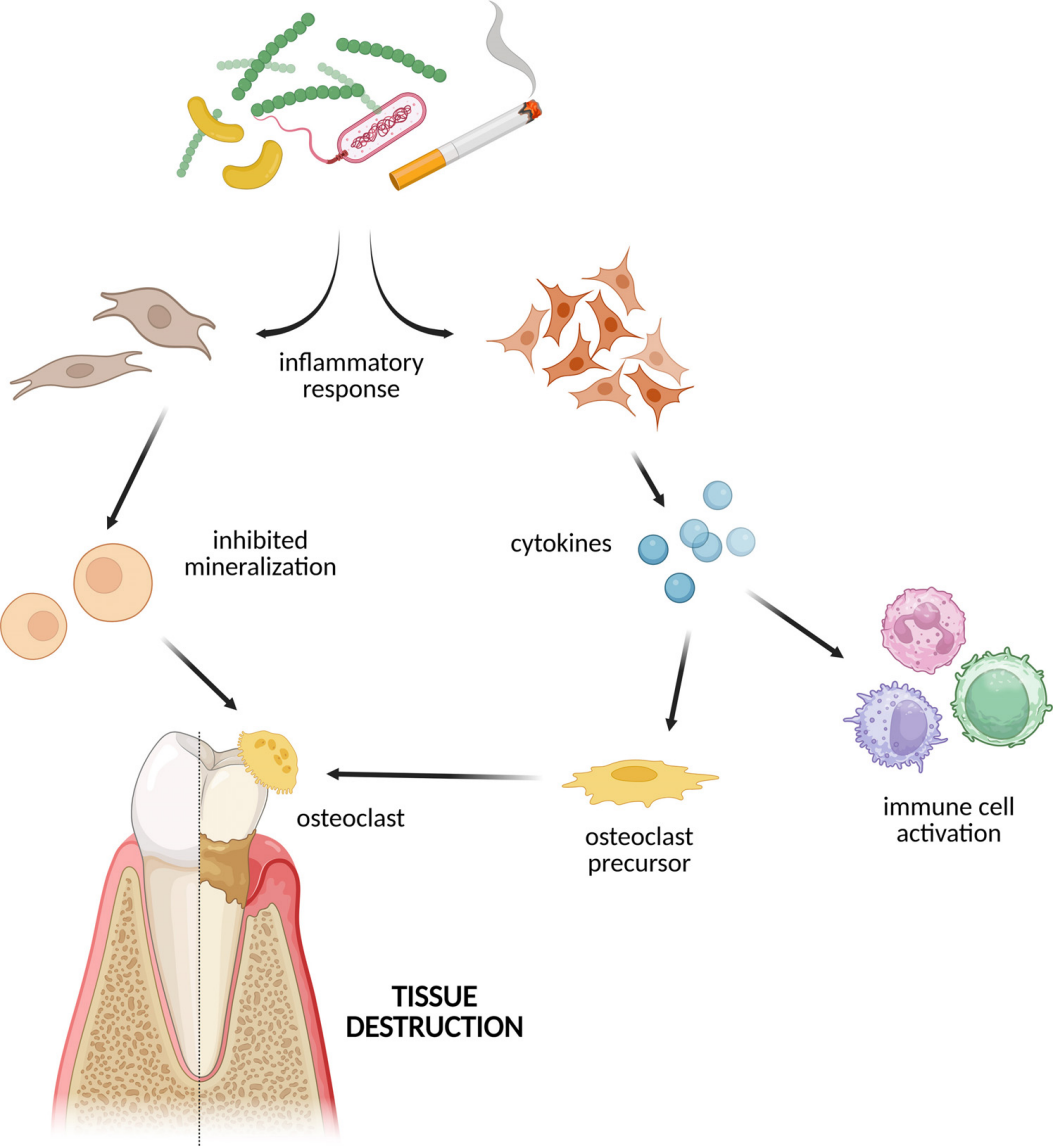
**Impact of lifestyle factors on epigenetic modifications through the selective modulation of the TLR signaling pathway, nicotine addiction pathway, transforming growth factor-β (TGF-β) signaling, and the hypoxia-inducible factor-1 (HIF-1) pathway.** Created with biorender.com.

Tobacco exposure, a significant environmental factor, has been demonstrated to substantially modify DNA methylation patterns [86]. Initially, cigarette smoke may disrupt this process by inducing DNA damage, which subsequently activates DNMTs [87]. Differential hypermethylation and hypomethylation of genes in response to smoking have been linked to smoking-related disorders, particularly inflammatory diseases. Notably, a significant association has been identified between the *HIVEP3* (Schnurri-3) gene and smoking, likely mediated by DNA methylation. *HIVEP3* is a recently characterized zinc finger protein that regulates osteoblast and osteoclast functions, inhibiting osteogenic differentiation and contributing to bone destruction [88]. Smokers tend to experience more advanced periodontitis, characterized by greater attachment loss and deeper periodontal pockets, compared to non-smokers or individuals who have quit smoking [89]. Oliveira et al. [90] identified distinct methylation patterns in the *IL-8* gene promoter region among smokers with chronic periodontitis, in contrast to healthy non-smokers. In another study, DNA methylation was assessed in the promoter regions of *TLR2* and *TLR4* in gingival tissues from healthy individuals, smokers, and non-smokers with chronic periodontitis. The *TLR4* promoters were predominantly unmethylated across all groups, whereas *TLR2* exhibited a mixed methylation pattern, showing a tendency toward methylation at CpG sites targeted by the Hhal enzyme [91]. An integrated transcriptomic and methylomic analysis of human gingival tissues revealed that genes associated with extracellular matrix (ECM) and extracellular structure organization displayed reduced expression levels in smokers, which correlated with increased DNA methylation compared to non-smokers [92]. A comprehensive evaluation of miRNA expression in PDL cells exposed to nicotine illustrated selective modulation of the TLR signaling pathway, nicotine addiction pathway, transforming growth factor-β (TGF-β) signaling, and the hypoxia-inducible factor-1 (HIF-1) pathway, in contrast to untreated PDL cells [93] (Figure 2).

### Integrative multi-omics approaches to understanding periodontitis etiology

Recent research has focused on identifying molecular signatures of periodontitis derived from various periodontal tissues to elucidate disease initiation, progression, and the complex pathophysiology associated with this condition. Diverse omics methodologies have been employed to systematically analyze the pathological processes associated with periodontitis [94]. One analysis indicated that the integration of multiple omics approaches enhances the differentiation of disease states and the assessment of treatment responses, particularly when metabolomic data is included. Additionally, multi-omics analyses of biofilm instability provide significant insights into the ecological dynamics that contribute to the progression of periodontal disease [95]. Shotgun metagenomics techniques have been utilized to explore the microbial communities linked to periodontitis. These methods offer a less biased characterization of microbial diversity, although they may be limited by lower sequencing depth. Importantly, metagenomics facilitates the functional profiling of microbial diversity by evaluating their gene content in relation to disease status. Evidence suggests that deeper periodontal pockets, indicative of more severe disease, display a higher prevalence of metabolic pathways and virulence factors, alongside a reduced representation of biosynthetic functions, when compared to shallower pockets in clinically healthy patients [96–98]. Richter et al. conducted an epigenome-wide association study (EWAS) to identify biologically active methylation marks in the oral masticatory mucosa [99]. These investigations aim to clarify the epigenetic mechanisms underlying the pathogenesis of periodontitis.

Furthermore, the expanding knowledge of genetic and epigenetic mechanisms associated with periodontal disease holds potential clinical relevance, particularly in the context of regenerative therapies. Recent studies suggest that tissue engineering approaches—such as utilizing stem cells and biologically active matrices—may benefit from targeting these molecular pathways to enhance periodontal healing and implant integration [100].

## Clinical and therapeutic implications

### Potential biomarkers for early diagnosis and risk assessment

Traditional biomarkers, including proteins, metabolites, and matrix metalloproteinases (MMPs), have been utilized in the diagnosis of periodontal disease. Additionally, epigenetic biomarkers, which modulate gene expression and its regulatory mechanisms, play significant roles in the onset and progression of periodontal disease. For example, chronic inflammation within the periodontium can disrupt DNA methylation patterns in genes encoding both pro-inflammatory (e.g., TNF-α and IL-6) and anti-inflammatory (e.g., IL-4 and IL-10) cytokines. Identifying these molecular and epigenetic biomarkers in oral biofluids offers promise for the early detection of periodontal disease during its initial stages of progression [101]. Cytokines and host-derived enzymes represent the most well-characterized emerging biomarkers, particularly concerning immune system activity [102]. The genetic architecture, defined as the composition of genetic variants underlying a specific trait, along with external factors such as lifestyle, influences the host’s ability to maintain the structural and immunological integrity of gingival tissues. Consequently, understanding genetic predisposition to periodontitis presents an opportunity for developing biomarkers to assess individual genetic risk [103].

A recent review suggests that specific salivary miRNAs have significant potential for diagnostic, prognostic, and therapeutic applications [104]. miRNAs are gaining recognition as promising biomarkers for periodontitis due to their regulatory roles in inflammation. Research has demonstrated that certain salivary miRNAs, such as hsa-miR-381-3p and miR-143-3p, are altered in patients with periodontitis and correlate with disease severity [105, 106]. Recent advancements in microfluidic and biosensor technologies facilitate the detection of these miRNAs in saliva, indicating the feasibility of non-invasive diagnostic platforms applicable in clinical settings. However, broader clinical application requires further validation across diverse populations and the establishment of standardized protocols to ensure consistent and accurate detection [107]. In addition to salivary biomarkers, periodontitis significantly alters specific miRNAs in gingival crevicular fluid, underscoring their role as local biomarkers involved in key regulatory pathways. Given their stability in oral fluids, these miRNAs have the potential to serve as non-invasive tools for the early diagnosis and monitoring of periodontal disease [108, 109].

**Figure 3.**
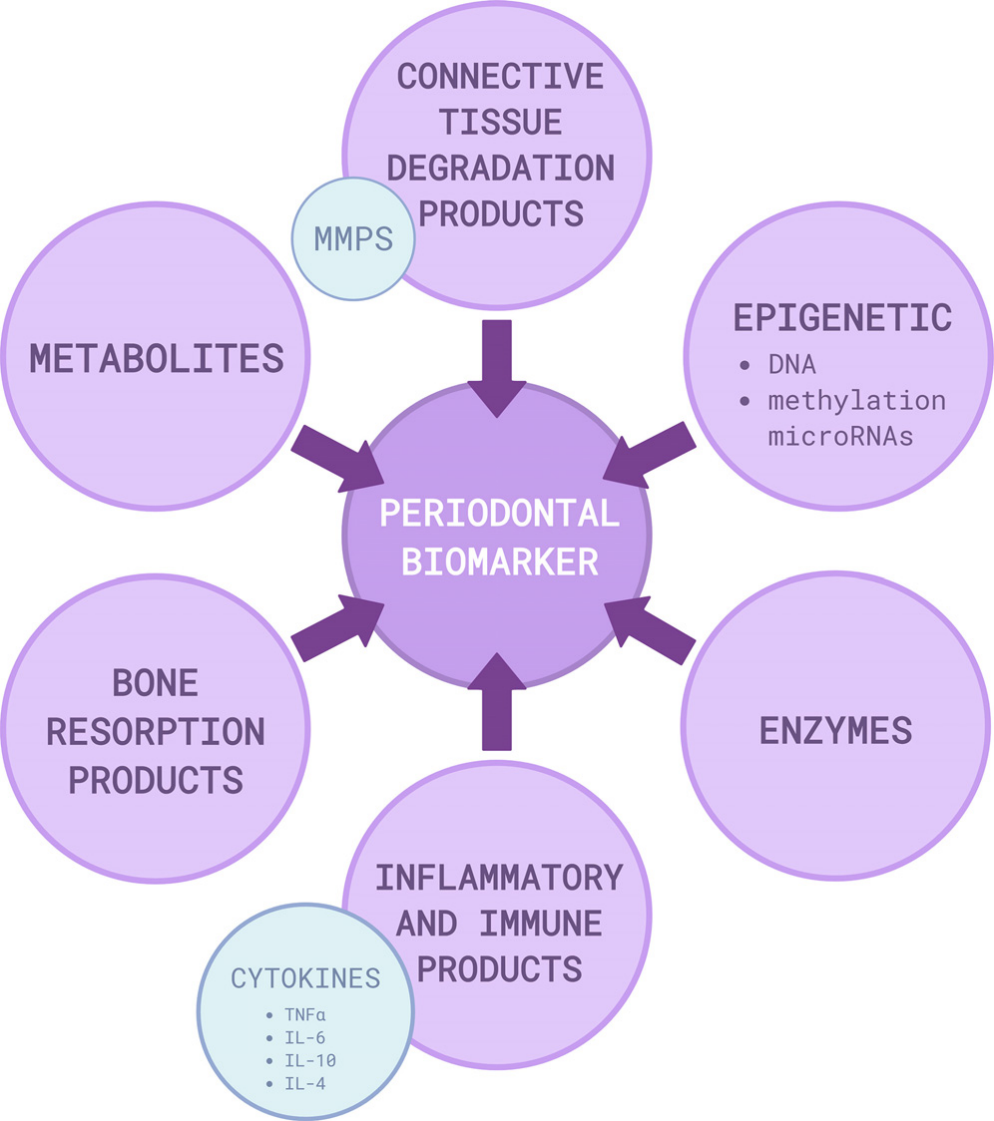
**Multi-omics integrative analysis approach that identifies gene-metabolite-pathway network involving several potential biomarkers for periodontitis during the early stages of disease.** Created with biorender.com.

MMP-8 is an enzyme primarily secreted by neutrophils and has been extensively investigated as a biomarker [110, 111]. MMP-8 and myeloperoxidase (MPO) exhibit the highest discriminatory potential between gingival disease and healthy tissue. The diagnostic utility of active MMP-8 (aMMP-8) in classifying periodontal disease, along with its application in point-of-care (POC) settings, has been rigorously examined, significantly impacting the classification frameworks for periodontitis and peri-implantitis [112]. Research indicates a correlation between protease and collagenase concentrations in gingival cervical fluid (GCF) and changes in periodontal pocket depth characteristic of periodontal disease. Barros and Offenbacher [46] highlighted that GCF serves as a reservoir for various biomarkers that can indicate disease presence, with the potential to differentiate between active and inactive periodontal sites. *CD163* has shown diagnostic potential for periodontal disease, with expression levels significantly elevated in diseased tissue compared to healthy tissue, thus identifying *CD163* as a promising biomarker for periodontitis [113]. Additionally, a multi-omics integrative analysis identified a gene-metabolite-pathway network involving *PDGFD*, *NRTN*, and *IL2RG* as potential biomarkers for periodontitis, suggesting that these factors may modulate disease progression through deoxyinosine and the ABC transporter pathway [114] (Figure 3).

### Epigenetic therapy and personalized medicine approaches

Emerging paradigms such as precision medicine and personalized medicine enhance predictive capabilities by facilitating detailed patient stratification and individualized therapeutic strategies. The implementation of these approaches necessitates the development of advanced diagnostic frameworks that integrate conventional clinical assessments with comprehensive data on patient-specific factors, including biomarkers, genetic profiles, environmental influences, and lifestyle characteristics [115]. By incorporating environmental and lifestyle factors and utilizing artificial intelligence (AI), precision medicine enables the identification of individual risk profiles and characteristics, thus facilitating the development of targeted prevention and treatment strategies [116]. Recent studies increasingly underscore the necessity for active involvement of researchers and oral health professionals in the integration of salivary diagnostic approaches. These methodologies demonstrate significant potential for the early detection of oral pathologies, enable more precise patient risk stratification, and support efforts to mitigate the global burden of oral diseases, particularly within the context of personalized medicine for conditions such as malocclusion, dental transposition, and periodontitis [117]. Bartold and Ivanovski [118] introduced a clinical model for periodontitis management known as P4 Periodontics, which embodies the principles of P4 medicine—prediction, prevention, personalization, and participation—as foundational elements of patient-centered care. The revised classification system aims to introduce a multidimensional diagnostic approach that accounts for individual risk factors, contrasting with the traditional method focused solely on periodontal tissue destruction [119]. This framework is designed to optimize patient care and advance the principles of precision and personalized medicine [115]. Individuals exhibiting epigenetic modifications related to pathologies often show limited responsiveness to standard therapeutic approaches. Consequently, personalized medicine strategies, including the use of targeted pharmacological agents tailored to the patient’s unique genomic profile, may offer more effective management of these conditions [120].

### Challenges and future perspectives in translating research into clinical practice

Examining alterations in DNA methylation provides valuable insights into the pathogenic mechanisms of periodontitis, potentially explaining why individuals with the same clinical subtype exhibit differing rates of disease progression and variable responses to standardized treatments. Future research should incorporate the analysis and reporting of lifestyle and environmental factors that may influence the emergence of epigenetic modifications at both cellular and tissue levels. Such studies may also facilitate the identification of consistent epigenetic markers indicative of individual susceptibility to disease progression, thereby advancing the development of precise and personalized therapeutic strategies for periodontitis.

Although current evidence indicates a role for epigenetic modifications in regulating gene expression, further research is necessary to clarify their specific impact on periodontal tissue degradation and their potential influence on the progression and severity of systemic conditions. A deeper understanding of these mechanisms will elucidate the broader implications of epigenetic regulation in both oral and systemic health.

## Conclusion

Insights from genome-wide studies on DNA methylation and genetic polymorphisms have enriched our understanding of how host genetic and epigenetic landscapes modulate immune and inflammatory responses, thereby contributing to periodontal tissue breakdown. These molecular alterations not only affect disease susceptibility and progression but also present opportunities for biomarkers that improve diagnosis and serve as targets for personalized therapeutic interventions. Advancing research through integrative multi-omics approaches will be crucial for unraveling the complex mechanisms underlying periodontitis and for translating these insights into precision diagnostic tools and targeted treatment strategies tailored to individual risk profiles. The combined analysis of genetic and epigenetic factors highlights the multifactorial nature of periodontitis, underscoring the importance of comprehensive approaches to disease characterization and management. Future research should prioritize translating these molecular insights into practical clinical applications, ultimately enhancing diagnostics and improving patient outcomes.
